# Palatopharyngeus muscle: the key in the pharyngoplasty surgeries for obstructive sleep apnea^☆^

**DOI:** 10.1016/j.bjorl.2019.04.001

**Published:** 2019-05-01

**Authors:** Luiz Ubirajara Sennes

**Affiliations:** a Universidade de São Paulo (USP), Faculdade de Medicina, Disciplina de Otorrinolaringologia, São Paulo, SP, Brazil; b Universidade de São Paulo (USP), Programa de Pós-Graduação em Otorrinolaringologia, São Paulo, SP, Brazil

Obstructive sleep apnea (OSA) is a highly prevalent disease about which we still have little knowledge. Its pathophysiology is complex, involving multiple anatomic and functional mechanisms. CPAP (Continuous Positive Airway Pressure) is considered the best treatment but has low adherence. Usage of CPAP for 4 h per night was considered adequate, but seems to be ineffective.

The uvulopalatopharyngoplasty (UPPP) which removes redundant pharyngeal tissues was widely used by otolaryngologists, but with inconsistent results. More recently, more aggressive pharyngoplasties have been described, that modify the muscular structure of the pharynx to achieve more stabilization.

The lateral pharyngoplasty (LP) described by Cahali (2003) was the first proposal for repositioning the lateral pharyngeal wall musculature in the treatment of OSA.^1^ Based in this new concept Pang & Woodson (2007) described the Expansion Sphincter Pharyngoplasty (ESP).^2^ In 2013, Sorrenti & Piccin proposed a conservative modification of ESP describing as Functional Expansion Pharyngoplasty (FEP).^3^ Cahali also improved the LP, which in version 6 seems to have reached its maturity.

Although there are no randomized studies comparing the results of LP and ESP/FEP,^4^ technical analysis may suggest some effects on pharyngeal patency during breathing.

Both LP and ESP/FEP promote the advancement of the soft palate and stabilization of the lateral wall, that can be observed by nasopharyngoscopy. The major difference between them is in the preparation and repositioning of the palatopharyngeus muscle (PPM).

In the LP the mucosa and the PPM are separated from the superior pharyngeus constrictor muscle (SPC) forming a thick and resistant muscle-mucosal flap with a superior and medial pedicle and without an inferior or posterior connection. After a small myotomy of SCP at the soft palate level, the PPM flap is repositioned and sutured anteriorly in a higher position to strengthen the lateral pharyngeal wall at the soft palate level.

In ESP/FEP the palatopharyngeus muscle is isolated from the mucosa and from SPC (partially) and transected inferiorly, forming a muscle flap with superior and medial pedicle. The free end of the PPM is rotated superoanterolaterally and sutured at the transition between soft and hard palate, below the mucosa. A suture of the remaining mucosal flap to the anterior tonsillar pillars covers the pharyngeal lateral wall.

In both techniques, the oropharynx is enlarged by removing the PPM that represents the main part of lateral pharyngeal bulk. Also, both eliminate the posteroinferior traction of the soft palate by PPM contraction.

In LP the flap ensures the healing of the lateral pharyngeal wall with the anterior tonsillar pillar, despite the necessity for healing of the posteroinferior aspect of the lateral pharyngeal wall relieve incision by secondary intention. Associated with the SPC myotomy, it creates a wider and more rectangular and stable retropalatal area.

In ESP/FEP the PPM acts by anchoring the soft palate anteriorly and creating lateral wall tension. It also enlarges and gives a rectangular shape to the retropalatal area. However, dehiscence of the lateral wall of the pharynx with the anterior tonsillar pillar is more critical, because it is covered only by a mucosal flap rather than muscle-mucosal flap as in LP.

In this sense LP prioritizes a thicker muscle-mucosal flap to reinforce the lateral pharyngeal wall, whereas ESP/FEP prioritizes the anterior anchorage of the soft palate and lateral pharyngeal wall traction, assuming greater risk of dehiscence of the thin mucosal flap.

To minimize these problem two maneuvers can be associated to ESP/FEP: 1. If PPM is a bulky muscle (which often occurs in the OSA patient), part of the PPM may be left adherent to the mucosa flap, creating a thicker and more resistant tissue. 2. If the suture in palatoglossus pillar creates tension in the mucosal flap, one or two relief incisions can be made in the posterior pharyngeal wall to facilitate adherence of mucosal flap to the lateral pharyngeal wall (similar to LP relieve incisions).

LP and ESP/FEP offer better results than UPPP, and have greater acceptance by surgeons. The question that remains is what happens with the PPM after its repositioning. Would this muscle develop a dilating action in the pharynx?

To have this answer we must know if the repositioned PPM preserves its function. The first aspect is whether its blood supply through the small pedicle is sufficient to maintain it as a muscular structure or only as fibrous tissue. Even in this condition, the PPM flap would be beneficial by promoting reinforcement in LP and anchorage into the ESP/FEP.

The second aspect is whether PPM motor innervation is preserved through the pedicle, maintaining a contractile function. If that occurs, contraction in LP would stiffen the lateral wall of the pharynx at the level of the soft palate, helping the pharyngeal support against collapse. In ESP/FEP its contraction would pull the soft palate anteriorly and superiorly, tensing the lateral pharyngeal wall and also favoring the opening and sustentation of the pharynx. In this sense, the constrictor function of PPM would become a dilating action of the pharynx during inspiration.

We still do not have studies in this sense. However, the progressive improvement of the surgical results and the aspect of the retropalatal area comparing early and late postoperative period may suggest that after an initial trauma this muscle recover its muscular function, acting as another dilator of the pharynx.

## Conflicts of interest

The author declares no conflicts of interest.
